# Efficacy and safety of stereotactic radiotherapy in patients with HER2-positive breast cancer brain metastases following intracranial oligoprogression

**DOI:** 10.3389/fonc.2026.1819733

**Published:** 2026-06-19

**Authors:** Shen Dongxing, Zhu Longyu, Zhang Jun, Kong Deyou, Liu Zhikun

**Affiliations:** Department of Radiation Oncology, The Fourth Hospital of Hebei Medical University, Shijiazhuang, Hebei, China

**Keywords:** brain metastases, breast cancer, HER2-positive, oligoprogression, stereotactic radiotherapy

## Abstract

Tyrosine kinase inhibitors (TKIs) demonstrate clear therapeutic effects in patients with HER2−positive breast cancer complicated by brain metastases. However, the efficacy and safety of salvage stereotactic radiotherapy (SRT) for intracranial oligoprogression after TKI therapy remain unclear. This single-center retrospective cohort study aimed to evaluate the clinical value of SRT in this specific population, enrolling 40 HER2-positive breast cancer patients with brain metastases who received SRT for intracranial oligoprogression after TKI therapy at the Department of Radiotherapy, Fourth Hospital of Hebei Medical University between January 2020 and January 2025. Patients were stratified into the MRI-stable disease (SD) group and MRI-progressive disease (PD) group according to intracranial oligoprogression features. The main outcome measures included post−radiotherapy intracranial progression−free survival (poRT−iPFS), time to next systemic treatment (NEST), overall survival (OS), and treatment safety. The median follow-up duration was 10.7 months (interquartile range [IQR] 7.0–20.8 months), with a median poRT-iPFS of 6.4 months (95% confidence interval [CI] 6.3–8.3 months), median NEST of 5.2 months (95% CI 4.7–6.5 months), and median OS of 9.5 months (95% CI 8.6–10.6 months). Stratified analysis revealed a significantly longer median poRT-iPFS in the MRI-SD group than in the MRI-PD group (7.9 months, 95% CI 7.5–8.3 vs. 5.5 months, 95% CI 4.6–6.4; P < 0.001). The main grade ≥ 3 treatment-related adverse events (TEAEs) were diarrhoea (20%), anaemia (20%), leucopenia (18%), nausea (15%), and thrombocytopenia (13%); Only 2 patients (5%) developed grade 1 radiation necrosis, and no interstitial pneumonia was reported. In conclusion, SRT provides considerable survival benefits and a manageable safety profile for HER2-positive breast cancer patients with brain metastases who develop intracranial oligoprogression after TKI therapy. MRI-stable intracranial lesions accompanied by increased peritumoural oedema may serve as a valuable indicator for initiating proactive radiotherapy intervention.

## 1 Introduction

Breast cancer ranks second only to lung cancer as the most common malignancy associated with brain metastasis (1). Statistics indicate that roughly one-third of breast cancer patients develop brain metastasis during the disease course, and this proportion reaches nearly half among those with the human epidermal growth factor receptor 2 (HER2)-positive subtype (2). Local radiotherapy and surgical resection are well-established standard treatments for breast cancer-related brain metastases (3). Nevertheless, more than half of patients develop local recurrence or new intracranial lesions within 12 months after radiotherapy (4), highlighting the unmet need for optimized treatment strategies. Tyrosine kinase inhibitors (TKIs) are small-molecule compounds that can cross the blood-brain barrier and exert distinct anti-neoplastic activity against intracranial lesions, making them a key systemic treatment for patients with breast cancer brain metastases (5), demonstrating superior efficacy compared to large-molecule monoclonal antibodies (6). The efficacy of TKIs such as neratinib, tucatinib, lapatinib, and pyrotinib has been confirmed in multiple prospective studies (7–10). In clinical practice in China, constrained by drug availability and treatment costs, the combination of TKIs such as pyrotinib or lapatinib with capecitabine remains one of the mainstream second-line treatment regimens for HER2-positive advanced breast cancer. For patients with stable or mildly symptomatic active brain metastases, initiating anti-HER2 therapy with close monitoring to defer radiotherapy is a common clinical strategy. However, the efficacy and safety of salvage radiotherapy following intracranial progression remain unclear. The present study did not compare deferred with concurrent stereotactic radiotherapy (SRT), but instead evaluated the efficacy and safety of SRT in patients with HER2-positive breast cancer brain metastases who developed intracranial oligoprogression after TKI therapy.

## 2 Methods

### 2.1 Study population

**Figure 1:**
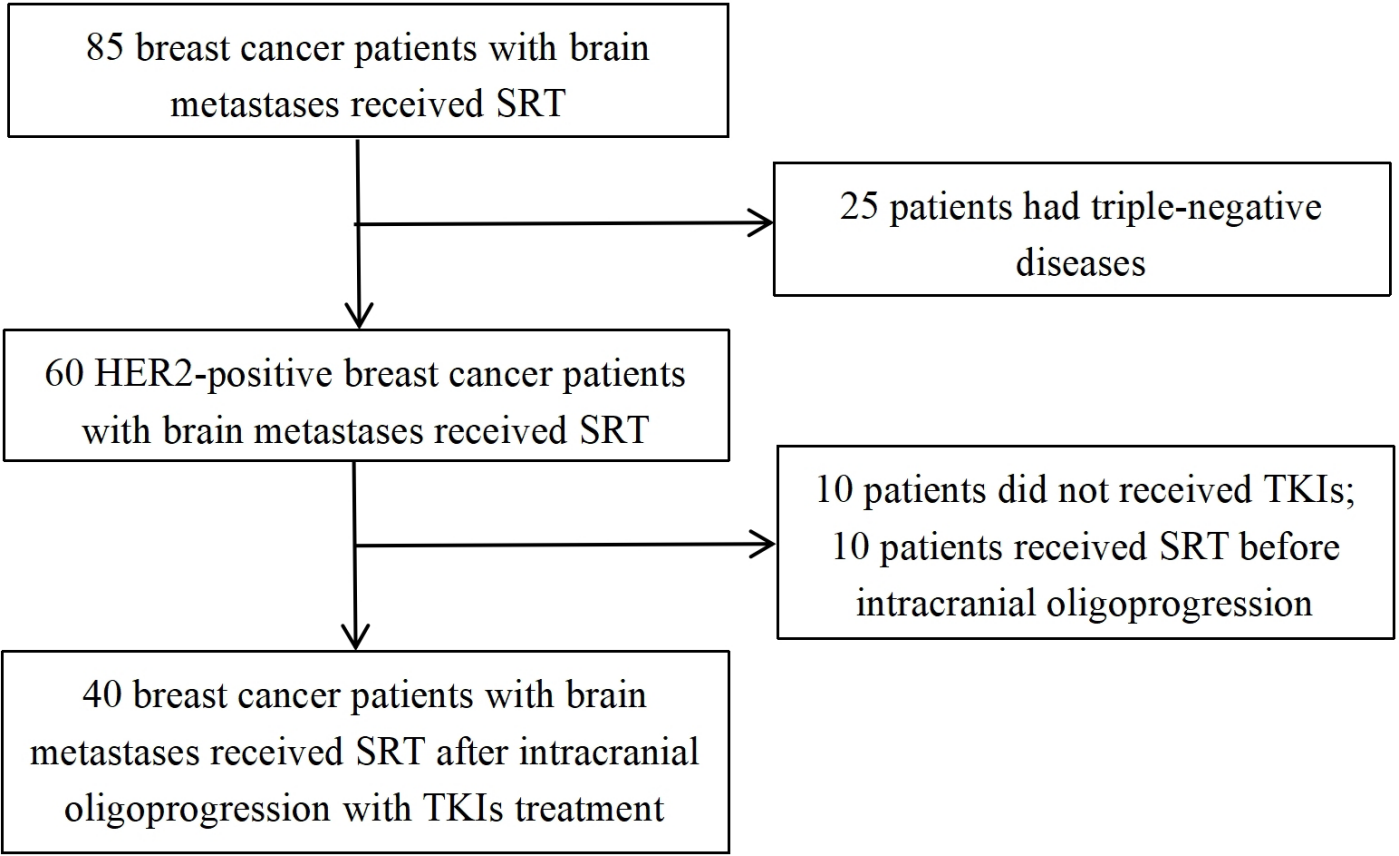
Study flow chart. Keys: SRT: stereotactic radiotherapy; HER2: human epidermal growth factor receptor 2; TKIs: tyrosine kinase inhibitors.

This was a single-centre retrospective cohort study. A total of 85 breast cancer patients with brain metastases who underwent SRT at the Department of Radiotherapy, Fourth Hospital of Hebei Medical University between January 2020 and January 2025 were initially screened. Of these, 60 patients were confirmed to be HER2-positive, and 45 received SRT for intracranial progression following TKI therapy. Intracranial oligoprogression was defined as ≤ 5 intracranial metastatic lesions with a maximum diameter ≤ 4.0 cm. After strict application of the predefined inclusion and exclusion criteria, 40 patients were finally included in the study cohort (**Figure 1**). Inclusion criteria: ① Female patients aged > 18 years; ② Eastern Cooperative Oncology Group (ECOG) performance status score ≤ 3, with life expectancy ≥ 3 months; ③ Pathologically verified HER2-positive and hormone receptor-negative breast cancer; ④ MRI-confirmed intracranial oligoprogression after TKI therapy; ⑤ ≤ 3 months interval between brain contrast-enhanced MRI follow-ups after metastasis;⑥ No treatment contraindications. Exclusion criteria: ① Pregnant or lactating women; ② Prior intracranial local therapy; ③ Concurrent leptomeningeal metastasis; ④ Presence of other malignancies. This study was approved by the Ethics Committee of the Fourth Hospital of Hebei Medical University (approval number: 2025KS031). Informed consent was waived in accordance with the committee’s regulations for retrospective studies.

### 2.2 Treatment methods

All treated patients received TKI therapy in combination with capecitabine. The TKI regimens were as follows: pyrotinib 400 mg once daily (21−day cycles), neratinib 240 mg once daily (21−day cycles), or lapatinib 1250 mg once daily (21−day cycles). Capecitabine was administered at 1000 mg/m² twice daily for 14 consecutive days, with a 21−day treatment cycle. SRT dose schedules included 19 Gy in 1 fraction, 20 Gy in 1 fraction, 24 Gy in 3 fractions, 27 Gy in 3 fractions, 27.5 Gy in 5 fractions, and 30 Gy in 5 fractions. Appropriate doses were selected according to guidelines for brain metastasis radiotherapy, based on lesion size and number. All patients received CyberKnife−guided SRT. For treatment planning, contrast−enhanced MRI was obtained within 14 days before the first fraction and co−registered with planning CT images to ensure accurate target localisation. The gross tumour volume (GTV) was delineated as visible metastatic lesions identified on both contrast−enhanced CT and MRI. For CyberKnife SRT, the planning target volume (PTV) was created by adding a 1−mm uniform margin to the GTV to account for set−up errors and tumour movement.

For patients with isolated intracranial oligoprogression, the original TKI regimen was continued during SRT. For three patients with extracranial progression (only 3 patients): SRT was delivered first. Considering the potential risk of increased radiation−induced brain necrosis associated with antibody−drug conjugates (ADCs), subsequent ADC treatment was initiated at 2 weeks after completion of SRT.

### 2.3 Endpoints

Follow-up was completed on 15 June 2025, with a 100% follow-up rate. The primary endpoint was post-radiotherapy intracranial progression-free survival (poRT-iPFS), defined as the interval from the start of SRT to the first documentation of intracranial disease progression, death from any cause, or the date of final follow-up. Secondary endpoints included time to next systemic treatment (NEST), post-brain metastasis progression-free survival (poBM-PFS), overall survival (OS), intracranial objective response rate (ORR), and treatment safety. NEST was defined as the time from initiation of SRT to disease progression, intolerance to systemic therapy requiring a change of regimen, or death from any cause. PoBM-PFS was defined as the interval from diagnosis of brain metastasis to disease progression prior to SRT. OS was defined as the time from SRT to death from any cause or last follow-up. Short-term efficacy outcomes included ORR and disease control rate (DCR). ORR was defined as the proportion of patients achieving complete response (CR) plus partial response (PR), and DCR was defined as the proportion of patients with CR, PR, or stable disease (SD).Safety assessments were based on clinical symptoms, laboratory tests, and imaging findings. Intracranial responses were evaluated using the Response Assessment in Neuro-Oncology-Brain Metastases (RANO-BM) criteria. Extracranial and overall responses were assessed according to the Response Evaluation Criteria in Solid Tumours (RECIST) 1.1. Treatment-related adverse events were graded using the Common Terminology Criteria for Adverse Events (CTCAE) version 5.0. For prognostic evaluation of brain metastases, the modified Breast-Graded Prognostic Assessment (GPA) index was adopted, which incorporates key factors including performance status, age, molecular subtype, number of intracranial lesions, and extracranial metastatic status. Intracranial oligoprogression was classified into two subgroups: a. MRI-stable disease (SD): enlargement of intracranial lesions <20%, accompanied by increased peritumoural oedema; b. MRI-progressive disease (PD): new intracranial lesions or enlargement of existing lesions ≥20%.

### 2.4 Statistical analyses

All statistical analyses were performed using SPSS software (version 26.0; IBM Corp., Armonk, NY, USA). Continuous variables were expressed as median and interquartile range (IQR), while categorical variables were presented as frequencies and percentages (%). Between-group comparisons of categorical data were conducted using the chi-square test or Fisher’s exact test, as appropriate. Survival curves were constructed using the Kaplan–Meier method, and differences in survival outcomes between groups were compared using the log-rank test. A two-sided P < 0.05 was considered statistically significant. All statistical tests were performed in accordance with standard biostatistical guidelines to ensure the reliability and validity of the results.

## 3 Results

### 3.1 Patient characteristics

A total of 40 female patients were enrolled. The median age at brain metastasis was 52 years (IQR 26–77). Five patients (12%) had stage IV disease at initial diagnosis. The median number of prior treatment lines before brain metastasis was 2 (IQR 1–3). Twenty-eight patients (70%) received trastuzumab plus pertuzumab. Thirty-two patients (80%) had poBM-PFS >6 months, and 25 patients (62%) had a Breast-GPA score >2.5. Thirty-two patients (80%) received ≥2 cycles of TKIs, including 27 (68%) treated with pyrotinib. Twenty-two patients (55%) were categorised as MRI-PD and 18 patients (45%) as MRI-SD. A single brain lesion was present in 18 patients (45%), and 24 patients (60%) had a maximum tumour diameter ≤ 2 cm. Concurrent liver metastasis was observed in 2 patients (5%). The most common SRT dose schedule was 19–20 Gy in 1 fraction (18 patients, 45%). The median planning target volume (PTV) was 13.2 cubic centimeter (cc). After intracranial progression, subsequent therapies included antibody-drug conjugates (ADCs), chemotherapy, salvage radiotherapy, and palliative surgical resection. Specifically, 10 patients (25%) received trastuzumab deruxtecan (T-DXd) and 12 patients (30%) received trastuzumab emtansine (T-DM1). Chemotherapy was administered to 20 patients (45%), salvage radiotherapy to 8 patients (20%), and palliative surgical resection to 4 patients (10%). (**Table 1**).

**Table 1:** Patient characteristics for the whole cohort (N=40).

Characteristic	N (%)	Characteristic	N (%)
Median age at BM (IQR)	52 (26–77)	Max diameter of BM	
Initial TNM staging		≥ 2 cm	16 (40)
Stage II	10 (25)	< 2 cm	24 (60)
Stage III	25 (63)	N of BM	
Stage IV	5 (12)	1	18 (45)
Median Line of therapy before BM, (IQR)	2 (1–3)	2–3	12 (30)
Anti-HER2 therapy before BM		4–5	10 (25)
Trastuzumab	10 (25)	Extracranial metastases	
Trastuzumab + Pertuzumab	28 (70)	Bone	8 (20)
Lapatinib	2 (5)	Lung	10 (25)
poBM-PFS		Liver	2 (5)
≤ 6 months	8 (20)	None	20 (50)
> 6 months	32 (80)	SRT dose	
Breast-GPA score		19 Gy/1F	8 (20)
≤ 2.5 points	15 (38)	20 Gy/1F	10 (25)
> 2.5 points	25 (62)	24 Gy/3F	2 (5)
TKI therapy duration		27 Gy/3F	2 (5)
≥ 2 cycles	32 (80)	27.5 Gy/5F	8 (20)
< 2 cycles	8 (20)	30 Gy/5F	10 (25)
TKIs category		Median volume of PTV(cc) (IQR)	13.2 (0.1–56.5)
Pyrotinib	27 (68)	Later-line therapy	
Neratinib	8 (20)	T-DXd	10 (25)
Lapatinib	5 (12)	T-DM1	12 (30)
Intracranial oligoprogression		Chemotherapy	20 (50)
MRI-SD	18 (45)	Salvage radiotherapy	8 (20)
MRI-PD	22 (55)	Palliative surgical resection	4 (10)

N, number; BM, brain metastases; IQR, interquartile range; HER2, human epidermal growth factor receptor 2; poBM-PFS, post-brain metastases progression-free survival; GPA, graded prognostic assessment; TKIs, tyrosine kinase inhibitors; SD, stable disease; PD, progressive disease; Max, maximum; SRT, stereotactic radiotherapy; Gy, gray; PTV, planning target volume; cc, cubic centimeter; F, fraction; T-DXd, trastuzumab deruxtecan; T-DM1, trastuzumab emtansine.

### 3.2 Efficacy

**Figure 2:**
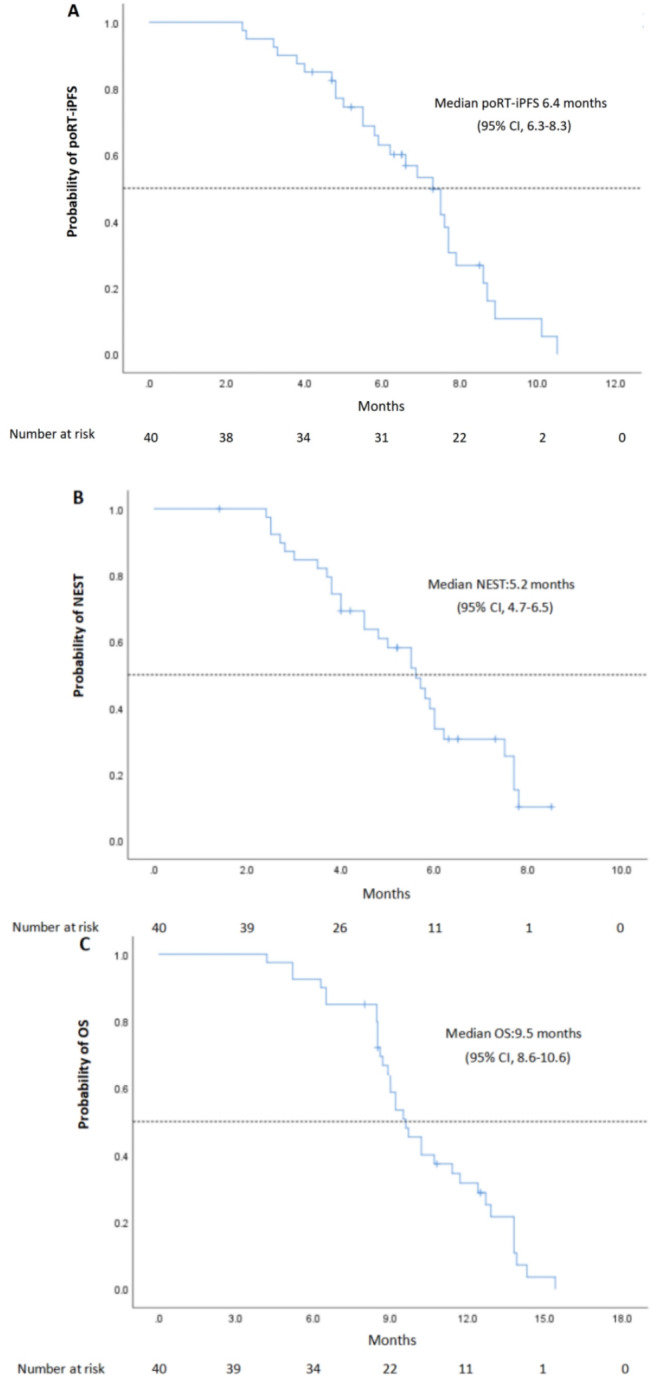
Kaplan-Meier analysis of post-radiotherapy intracranial progression-free survival **(A)**, time to next systemic treatment **(B)** and overall survival **(C)** in all patients (n = 40). Keys: CI, confidence interval; poRT-iPFS, post-radiotherapy progression-free survival; NEST, time to next systemic treatment; OS, overall survival.

Median follow-up was 10.7 months (IQR 7.0–20.8). As of 15 June, 2025, 5 patients (12%) remained alive and received T-DXd as later-line therapy. Median values were as follows: poRT-iPFS 6.4 months (95% CI 6.3–8.3), NEST 5.2 months (95% CI 4.7–6.5), OS 9.5 months (95% CI 8.6–10.6), and poBM-PFS 10.3 months (95% CI 7.1–11.5). The 6-month rates of poRT-iPFS and NEST were 85.0% and 33.7%, respectively, and the 10-month OS rate was 45.4%. (**Figure 2**).

The MRI-SD group showed significantly longer median poRT-iPFS than the MRI-PD group (7.9 vs. 5.5 months, P < 0.001). Median NEST was 6.0 months in the MRI-SD group and 5.0 months in the MRI-PD group (P = 0.058). Median OS was 9.0 months and 10.2 months, respectively (p=0.563). Subgroup analyses revealed no significant differences in poRT-iPFS, NEST, or OS among different TKI types or SRT dose groups (all P >0.05)(**Table 2**). Use of ADCs as salvage therapy after SRT did not affect poRT-iPFS or NEST, but was associated with longer median OS (11.2 vs. 9.0 months, P = 0.012).

**Table 2:** Survival analysis of different subgroups.

Subgroups	poRT-iPFS(months)			NEST(months)			OS(months)		
	Median (95%CI)	χ²	p-value	Median (95%CI)	χ²	p-value	Median (95%CI)	χ²	p-value
oligoprogression		23.147	<0.001		3.604	0.058		0.335	0.563
MRI-SD	7.9(7.5–8.3)			6.0(5.4–6.6)			9.0(8.7–10.3)		
MRI-PD	5.5(4.6–6.4)			5.0(3.8–6.2)			10.2(7.7–12.7)		
TKIs category		6.841	0.091		4.010	0.135		0.132	0.724
Pyrotinib	6.5(5.5–7.7)			5.0(5.0–6.9)			10.3(8.8–11.3)		
Others	6.2(5.0–7.4)			5.5(4.0–6.8)			9.1(8.1–10.5)		
SRT dose		3.214	0.073		1.983	0.159		2.756	0.097
19-20Gy/1F	6.0(4.5–7.3)			4.9(5.4–6.5)			9.3(7.7–11.3)		
24-27Gy/3F	6.5(4.6–8.4)			5.5(5.0–6.2)			9.2(7.2–10.6)		
27.5–30 Gy/5F	6.3(4.6–8.3)			5.0(4.3–7.2)			9.8(7.9–12.5)		

poRT-iPFS, post-radiotherapy progression-free survival; NEST, time to next systemic treatment; OS, overall survival; SD, stable disease; PD, progressive disease; CI, confidence intervals; χ², chi-square; TKIs, tyrosine kinase inhibitors; SRT, stereotactic radiotherapy; F, fraction.

At 3 months after SRT, the intracranial ORR and DCR were 65% and 88%, respectively. The ORR and DCR were 89% and 94% in the MRI-SD group, and 46% and 82% in the MRI-PD group.

### 3.3 Safety

Treatment-related adverse events (TEAEs) were evaluated in all 40 enrolled patients, with the majority being grade 1–2 and manageable with symptomatic treatment. The most commonly reported TEAEs included diarrhoea, leucopenia, anaemia, thrombocytopenia, and elevated alanine transaminase (ALT)/aspartate transaminase (AST). Grade ≥ 3 TEAEs occurred in 18 patients (45%), with the most frequent being diarrhoea (20%), anaemia (20%), leucopenia (13%), nausea (15%), and thrombocytopenia (13%). No grade 4 or 5 TEAEs were reported. Radiation-related adverse events were rare: only 2 patients (5%) developed grade 1 radiation necrosis. No cases of interstitial pneumonia or severe neurological complications were observed during follow-up. (**Table 3**).

**Table 3:** Treatment-related adverse events.

Adverse event, N (%)	Any grade	≥ Grade 3
Diarrhoea	38 (95)	8 (20)
Leucopenia	35 (88)	7 (18)
Anaemia	28 (70)	8 (20)
Thrombocytopenia	24 (60)	5 (13)
Elevated ALT or AST	20 (50)	0 (0)
Nausea	10 (25)	6 (15)
Vomiting	8 (20)	3 (8)
Fatigue	8 (20)	0 (0)
Rash	4 (10)	0 (0)
Radiation necrosis	2 (5)	0 (0)

N, number;AST, aspartate aminotransferase; ALT, alanine aminotransferase.

## 4 Discussion

Recent advances in anti−HER2 targeted therapy have substantially improved the prognosis of HER2−positive breast cancer patients with brain metastases. Consistent with this, Chinese guidelines recommend prioritising systemic therapy over immediate radiotherapy for patients with stable intracranial disease, aiming to delay radiation−induced neurotoxicity and preserve quality of life (11). While international guidelines now favour T-DXd as the preferred second-line treatment for these patients (12), TKIs remain the standard of care in many clinical settings, including China, due to accessibility and cost considerations. This study focuses on SRT strategies for intracranial oligoprogression following TKI therapy.

Studies such as PHENIX, PHOEBE, and PICTURE have demonstrated that pyrotinib confers favourable PFS benefits in advanced breast cancer (13–15), and the PERMEATE study confirmed its efficacy in patients with brain metastases (10). The BROPTIMA study further showed that pyrotinib combined with radiotherapy improved PFS (16), while other TKIs have also exhibited activity in HER2-positive breast cancer brain metastases (7, 9). However, the efficacy and safety of SRT following TKI failure remain unclear. Our study enrolled 40 patients with HER2-positive breast cancer brain metastases who underwent SRT for intracranial oligoprogression after TKI therapy. With a median poRT-iPFS of 6.4 months, median OS of 9.5 months, intracranial ORR of 65%, and DCR of 88%, the findings confirm that SRT combined with continued TKI therapy delivers substantial intracranial disease control and survival benefits. This provides a valuable therapeutic alternative for intracranial progression after TKI failure in Chinese clinical practice.

A key criterion for defining oligometastatic disease is the feasibility of safe, radical local radiotherapy for all detected lesions (17). A multicentre randomised phase II trial of 43 patients with oligometastatic breast cancer (≤ 5 lesions) showed that 21 patients receiving systemic therapy plus local treatments (radiotherapy/ablation) achieved 100% local control (18). A prospective–retrospective study of 129 patients with oligoprogressive breast cancer (≤ 5 lesions) reported a median poRT-iPFS of 11.3 months with systemic therapy plus SBRT (19), supporting the role of local radiotherapy for oligometastatic disease. Although breast cancer brain metastases are often excluded from such studies, some define intracranial oligometastasis as < 4 lesions (20). A meta-analysis noted that pre-treatment peritumoural oedema correlates with increased local failure risk after stereotactic radiosurgery (21). Integrating clinical practice and brain metastasis imaging characteristics, we defined intracranial oligoprogression as ≤ 5 lesions with a maximum diameter ≤ 4.0 cm, stratified into MRI-SD and MRI-PD groups. The median poRT-iPFS was 7.9 months vs. 5.5 months (P < 0.001), suggesting that MRI-stable lesions with increased peritumoural oedema represent a valuable imaging signal for prompt radiotherapy intervention under close surveillance and limited intracranial tumour burden.

For advanced breast cancer patients with oligoprogression, timely SRT may delay the need for systemic therapy escalation (22). An Italian prospective–retrospective study reported a median NEST of 13.6 months in patients with oligoprogression (≤ 5 lesions) treated with local radiotherapy plus systemic therapy (19), with comparable outcomes in another retrospective study (23). These findings were further validated in a phase II prospective trial (24) and the COMBART study (25). For subsequent salvage therapy, the DESTINY-Breast 03 phase 3 trial showed that T-DXd significantly improved PFS (28.8 months) and ORR (78.5%) in HER2-positive advanced breast cancer (26), and a retrospective study reported a median systemic PFS of 7.4 months with T-DXd in patients with brain metastases progressing after pyrotinib-based therapy (27). While our study reflects real-world Chinese population characteristics, its overall efficacy is inferior to these reports, which may be attributed to the low proportion of patients receiving ADCs in later lines.

The most common grade ≥ 3 TEAEs were diarrhoea (20%), anaemia (20%), leucopenia (18%), nausea (15%), and thrombocytopenia (13%), all related to TKI therapy. Regarding radiation-specific toxicity, two patients (5%) developed grade 1 radiation necrosis, with no severe neurological complications. This acceptable safety profile is consistent with previous studies (14, 28, 29).

This study has inherent limitations. First, the retrospective design is susceptible to selection and information bias. Second, the small sample size may limit statistical power. Third, neurocognitive function and quality of life were not assessed, restricting the comprehensiveness of outcomes. Finally, the low proportion of patients receiving ADCs may have impacted survival outcomes.

## 5 Conclusion

SRT provides favourable survival outcomes and a manageable safety profile in patients with HER2−positive breast cancer brain metastases who develop intracranial oligoprogression following TKI therapy. MRI−stable intracranial lesions accompanied by increased peritumoural oedema may serve as a valuable indicator for early proactive radiotherapy intervention.

## Data Availability

The raw data supporting the conclusions of this article will be made available by the authors, without undue reservation.
